# Cerebellar Contribution to Pattern Separation of Human Hippocampal Memory Circuits

**DOI:** 10.1007/s12311-015-0726-0

**Published:** 2015-10-06

**Authors:** Ayano Shiroma, Masahiko Nishimura, Hideki Nagamine, Tomohisa Miyagi, Yohei Hokama, Takashi Watanabe, Sadayuki Murayama, Masato Tsutsui, Daisuke Tominaga, Shogo Ishiuchi

**Affiliations:** 1 Department of Neurosurgery, Graduate School of Medicine, University of the Ryukyus, 207 Uehara, Nishihara-machi, Nakagami-gun, Okinawa 903-0215 Japan; 2 Department of Radiology, Graduate School of Medicine, University of the Ryukyus, 207 Uehara, Nishihara-machi, Nakagami-gun, Okinawa 903-0215 Japan; 3 Department of Pharmacology, Graduate School of Medicine, University of the Ryukyus, 207 Uehara, Nishihara-machi, Nakagami-gun, Okinawa 903-0215 Japan; 4 Okinawa Study Center, The Open University of Japan, 1Senbru, Nishihara, Okinawa 903-0219 Japan

**Keywords:** Posterior lateral cerebellum, Cognitive function, Brain tumor, Memory, Hippocampus, fMRI

## Abstract

The cerebellum is a crucial structure for cognitive function as well as motor control. Benign brain tumors such as schwannomas, meningiomas, and epidermoids tend to occur in the cerebellopontine angle cisterns and may cause compression of the posterior lateral cerebellum near the superior posterior fissure, where the eloquent area for cognitive function was recently identified. The present study examined cognitive impairment in patients with benign cerebellar tumors before and after surgical intervention in order to clarify the functional implications of this region in humans. Patients with cerebellar tumors showed deficits in psychomotor speed and working memory compared with healthy controls. Moreover, these impairments were more pronounced in patients with right cerebellar tumors. Functional magnetic resonance imaging during performance of a lure task also demonstrated that cerebellar tumors affected pattern separation or the ability to distinguish similar experiences of episodic memory or events with discrete, non-overlapping representations, which is one of the important cognitive functions related to the hippocampus. The present findings indicate that compression of the human posterior lateral cerebellum affects hippocampal memory function.

## Introduction

Recent research has shown that the cerebellum plays an important role in cognitive and emotional functions as well as motor control [1–4]. Studies using functional magnetic resonance imaging (fMRI) have shown that the actual use of a tool (e.g., scissors) primarily activates the anterior cerebellum, whereas imaginary use of a tool activates the lateral posterior lobe of the cerebellum [5]. Skilled use of the tool after learning activates a specific area near the superior posterior fissure, indicating that the posterior cerebellum is essential for information processing, space representation, and some procedural memory [6, 7] and is dependent upon the sequential relationship between discrete elements, just as in the serial reaction task. However, the acquisition of other skills does not require the learning of sequences like prism adaptation, which can be acquired during short-term motor learning. Human studies have identified the important contribution of the cerebellum to intrinsic functional connectivities [8, 9] and higher cognitive functions, especially to episodic memory, working memory, and procedural memory [10–13]. Nonetheless, the relationship between the cerebellum and hippocampal circuits in memory systems has not been fully evaluated.

Benign cerebellar tumors are isolated focal lesions that are frequently localized in the cerebellopontine angle or around the superior posterior fissure and do not invade or destroy neural networks, unlike gliomas or vascular strokes. These tumors are essentially regional, thereby allowing a more discrete estimation of the functionality of a specific region through examination of whether the function lost before operation recovers after surgical treatment. However, although preservation of VIIth and VIIIth cranial nerve function is prioritized during neurosurgical treatment, the cognitive function of these patients has not been evaluated. We therefore analyzed the cognitive impairment of patients before and after surgical intervention in order to evaluate the functional involvement of the posterior lateral cerebellum near the superior posterior fissure. We found that patients with right cerebellar tumors exhibited disturbances in psychomotor speed as examined by the digit symbol test (DST) and working memory as examined by the digit span test (DS) when compared with healthy controls. Nonetheless, the classical neuropsychological domain does not really have a distinct functional anatomy.

In the current study, we analyzed the distinct human cerebellar contribution to memory systems under whole brain network organization using the method of Global Brain Connectivity (GBC). Several past studies have indicated that cognition involves large-scale human brain systems with multiple interacting regions. We therefore tried to identify a prominent feature of this hub of human cognition using lure task-related and resting-state functional MRI (rs-fMRI) data. We focused on the pattern separation ability that discriminates between similar experiences of episodic memory, a crucial component of the hippocampal memory circuit, and used functional MRI (fMRI) to investigate subjects performing an established lure task [14]. Interestingly, patients with cerebellar tumors selectively showed a decreased ability for pattern separation in the lure task. We first identified nine regions related to pattern separation ability by imposing stringency criteria based on an activation map of lure task fMRI findings from normal volunteers. Blood oxygen level-dependent (BOLD) signals, which are one of the indices of hemodynamic responses to neural activity, were correlated to correct response rates in the lure task associated with the activity of the following four distinct regions: right and left cerebellar hemisphere (lobule VI/Crus I), left anterior mid-cingulate cortex (aMMC), and right hippocampal dentate gyrus (DG). We then tested whether these regions showed high GBC in the rest of the brain using rs-fMRI data. We found that GBC correlated to a correct response rate in the lure task was limited to three of these regions, excluding the left cerebellar lobule VI/Crus I. Finally, we ascertained that this correlation was altered in patients with right and left cerebellar tumors as compared with normal healthy volunteers. We therefore hypothesized that the cerebellar contribution to pattern separation ability is dependent upon integration of the right cerebellar hemisphere (lobule VI/Crus I) associated with the left aMMC and right hippocampal DG. The pattern changes in the functional connectivity of patients with cerebellar tumors may indicate an important contribution of the human cerebellum to higher cognitive functions associated with hippocampal memory systems.

## Materials and Methods

### Subjects

**Fig. 1:**
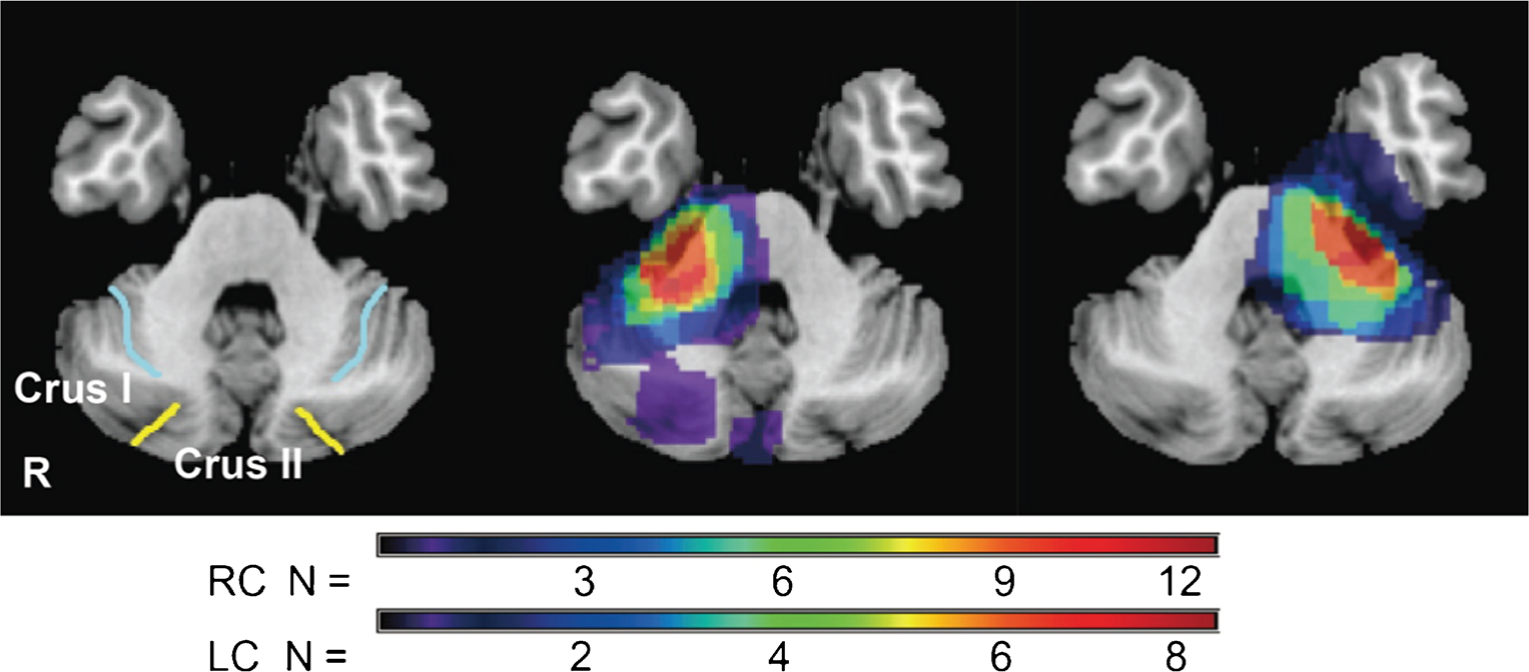
Tumor topography of right (*middle image*, *n* = 17) and left cerebellar tumors (*right image*, *n* = 11). *Light blue line* indicates the superior posterior fissure; *yellow line* indicates the horizontal fissure in the left image

Neuropsychological assessments were carried out on 28 patients with benign cerebellar tumors (mean age 50.9 ± 12.1 years; 11 males, 17 females), 17 with right cerebellar lesions (mean age 49.4 ± 13.6 years; 8 schwannomas, 8 meningiomas, and 1 epidermoid) and 11 with left cerebellar lesions (mean age 53.5 ± 8.3 years; 6 schwannomas, 2 meningiomas, 2 epidermoid, and 1 lipoma) (Table 1), as well as on a control group consisting of 23 healthy controls matched for age, sex, and years of education (mean age 53.4 ± 14.1, range 21–72 years; 9 males, 14 females). Regarding clinical histories, one patient (R19) had previously undergone gamma knife radiosurgery, and two patients (L10 and L17) had recurrent tumors. Patients were excluded for the following reasons: age under 20 or over 78 years; lesions involving non-cerebellar cortical or subcortical regions; history of alcohol or drug abuse; or pre-existing psychiatric disease. Neurological examinations of gait, kinetic function-arm, kinetic function-leg, speech, and eye movements were conducted based on the Brief Ataxia Rating Scale [15]. All patients showed normal performance except for two cases (R2 and R7) who walked almost naturally, but were unable to walk with their feet in the tandem position. The locations of the cerebellar tumors are shown in Fig. 1 and Table 2. Notably, the tumor compressed the posterior lateral cerebellum in all patients, especially lobule VI and Crus I (Fig. 1 and Table 2). Lesion size was measured in milliliters on preoperative MRI, according to the formula; *a* × *b* × *c* / 2, where *a* and *b* indicate the longest crossed dimension of the horizontal plane, and *c* indicates the greatest length of the tumor in the coronal plane.

**Table 1:** Summary of 28 patients examined by neuropsychological assessment

Patient number	Sex/age/handedness	Diagnosis	Lesion side	Size of lesion (mL)	Follow-up assessment	fMRI study (preop)
R1	F/38/right	Meningioma	Right	3.41		
R2	M/21/right	Schwannoma	Right	50.56	+	
R6	M/61/right	Schwannoma	Right	0.27	+	
R7	F/42/right	Schwannoma	Right	18.31	+	+
R10	F/51/right	Meningioma	Right	6.55	+	+
R11	M/64/right	Epidermoid	Right	2.66	+	+
R12	F/34/right	Meningioma	Right	3.46	+	+
R13	F/52/right	Meningioma	Right	5.85	+	
R14	M/63/right	Schwannoma	Right	7.95		+
R15	M/76/right	Schwannoma	Right	5.79		
R16	F/50/right	Meningioma	Right	5.79	+	+
R17	M/49/right	Schwannoma	Right	3.68	+	+
R18	F/40/right	Schwannoma	Right	11.7	+	+
R19	M/60/right	Schwannoma	Right	5.22		+
R20	F/40/right	Meningioma	Right	0.42	+	+
R21	F/36/right	Meningioma	Right	7.71	+	+
R22	F/63/right	Meningioma	Right	13.51		+
L2	M/49/right	Meningioma	Left	3.82		
L4	F/48/right	Schwannoma	Left	27.34		+
L9	F/55/right	Schwannoma	Left	3.18		
L10	F/47/right	Schwannoma	Left	4.69		+
L11	F/69/right	Meningioma	Left	5.68		+
L12	F/59/right	Epidermoid	Left	3.97		
L13	M/53/right	Lipoma	Left	0.79		+
L14	M/38/left	Schwannoma	Left	25.94		
L15	F/62/right	Schwannoma	Left	0.25		+
L16	M/55/right	Epidermoid	Left	13.55		+
L17	F/38/right	Schwannoma	Left	16.89		+

“+” indicates participation in the follow-up assessment or fMRI study

*M* male, *F* female

**Table 2:**
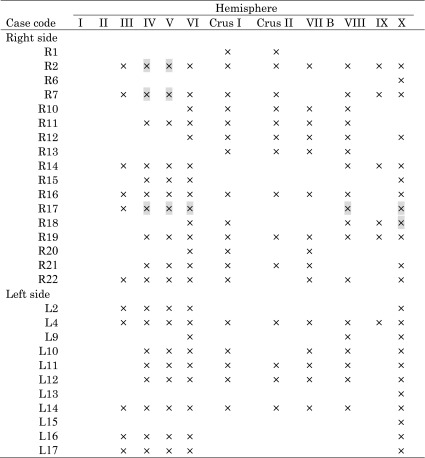
Lesion characteristics in patients with cerebellar tumors

“×” denotes the existence of a tumor at preoperative stage. “” indicates the residue at postoperative stage

### Informed Consent and Approval

All patients and control subjects provided written informed consent for this investigation. The study was approved by the ethical committee of the University of the Ryukyus.

### Experimental Design

In the preoperative stage, 28 patients with cerebellar lesions underwent neuropsychological assessments and 19 patients participated in the fMRI examination. The fMRI study was conducted once before surgical treatment. In the postoperative stage, 12 patients with right cerebellar tumors (mean age 45.0 ± 11.5 years, range 21–64 years; 3 males, 9 females) underwent follow-up neuropsychological assessments in order to examine whether surgical intervention had an effect on functional recovery. Detailed individual profiles are shown in Table 1.

### Neuropsychological Assessment

The battery consisted of the following tests: (I) mini-mental state examination (MMSE) [16] and modified MMSE (3MS) [17] for global cognitive screening, (II) Trail Making Test (TMT) [18] and Stroop test (ST) [18] for executive function, (III) Wechsler Adult Intelligence Scale-Revised (WAIS-R) digit span subtest (DS) [19] for working memory, (IV) WAIS-R DST [19] for psychomotor speed, (V) partial WAIS-R block design subtest (fifth and ninth items) [19] and the cube-copying test for visuospatial ability. For quantitative assessment of constructional ability in the cube-copying test, the points of connection and plane-orientation errors were evaluated. A point of connection was defined as a point at which three lines met to form a vertex, hence subjects could score up to eight points, since eight points of connection are present in a cube. Each plane with two pairs of parallel lines was evaluated in terms of the number of lines and the extent to which they were parallel. No plane-error points were scored if the cube was copied accurately [20]. We selected brief neuropsychological tests that could be performed within 1 h in order to reduce the burden on patients in the preoperative or postoperative therapeutic stage. As for the duration of patients’ follow-up, we carried out of the assessment within 6 months after resection of the tumor. Patient R2 with a huge schwannoma showed transient neurological symptoms related to the IVth nerve. The double vision by such nerve injury influenced cognitive performance, so we followed up the patient until recovery of its symptom.

### Event-Related fMRI Study

#### Subjects

Twelve patients with right cerebellar tumors (mean age 46.9 ± 13.3 years, range 17–66 years; 3 males, 9 females), 7 patients with left cerebellar tumors (mean age 53.3 ± 10.1 years, range 38–69 years; 2 males, 5 females), and 30 normal healthy volunteers (mean age 24.0 ± 5.2 years, range 22–35 years; 21 males, 9 females) were enrolled in this study (Table 1). The normal healthy volunteers that participated in the fMRI study were different from those included in the neuropsychological analysis. Standard values in each generation of the correct response rate in fMRI behavioral task were not established. Therefore, to estimate a normal value of the correct response rate, we recruited healthy young subject. None of these patients had any signs or history of neurological or psychological diseases. This study was approved by the ethical committee of the University of the Ryukyus with written informed consent obtained from all participants. Subjects were all right-handed according to the Edinburgh Handedness Laterality Index, with a median score of 100 (range 80–100).

#### Experimental Paradigm

**Fig. 2:**
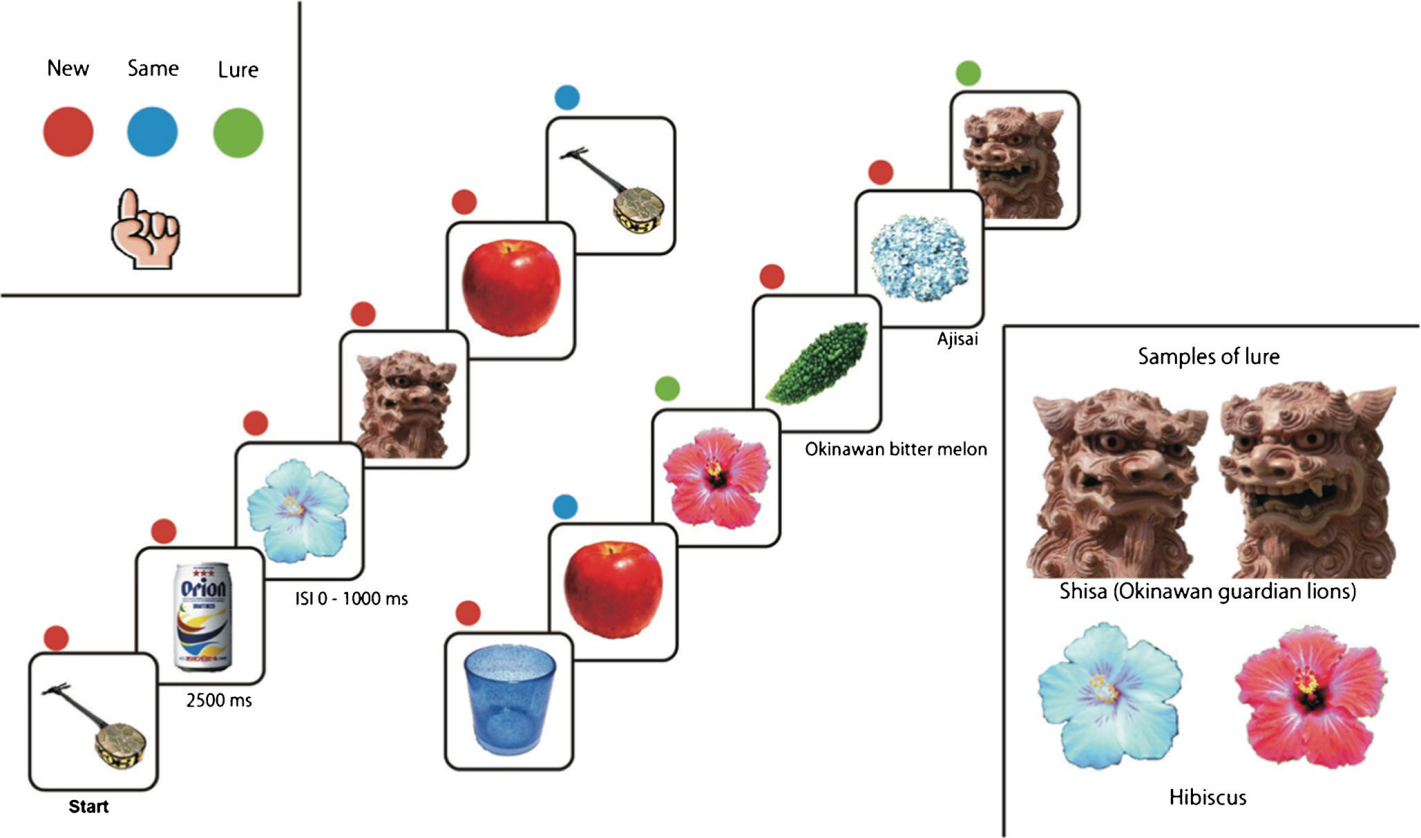
fMRI behavioral task. Images of single items were presented for 2,500 ms followed by a 0–1,000 ms interstimulus interval. Novel, repeated, and similar lure items were randomly shuffled in the task. *Upper left insets* show three task buttons for new (*red*), lure (*green*), and repeated (*blue*) stimuli. *Lower right insets* show examples of lure pairs; Okinawa guardian lions and hibiscuses

The fMRI behavioral paradigm used was a rapid event-related fMRI design [14, 21, 22] based on an explicit three-alternative forced choice task including novel (new), repeated (same), and lure (similar) stimuli consisting of color photographs of common objects. A fully randomized functional run consisted of 108 total trials, 16 lure sets, 16 repeat sets, and 44 unrelated novel items (foils) (Fig. 2). Forty-four foil trials, 16 trials first presented from repeat sets, and 16 trials first presented from lure sets were presented as the new stimuli. The same stimuli were 16 trials, which are second presented from repeat sets. The lure stimuli were 16 trials which are second presented from similar sets. Each stimulus was presented for 2,500 ms with a 0–1,000 ms interstimulus interval to prevent adaptive stimulus responses. The number of trials separating similar and identical pairs was randomly varied from 10 to 40 trials. Several photographs were displayed to participants on a goggles display during the session. If the photograph was first presented in the session, participants were required to press the *red* button indicating a new object. If the photograph had been displayed before in the session, examinees were instructed to press the *blue* button indicating a repeated object. Finally, if they thought that the photograph was similar to, but not the same as previous stimuli, they were required to press the *green* button, indicating a similar but not identical object. Responses and reaction times were recorded in a button box (Current Designs, Inc., Philadelphia, Pennsylvania). Visual stimuli were presented to the subjects using 800 × 600 resolution magnet-compatible goggles under computer control (Resonance Technologies, Inc., Salem, Massachusetts) using Presentation® software (Neurobehavioral Systems, Inc., Austin, Texas).

#### MRI Data Acquisitions

Anatomical and functional images were obtained using a 3-T MRI scanner (Discovery MR750; GE Medical System, Waukesha, Wisconsin, USA) with a 32-channel head coil and high-order manual shimming to the temporal lobes. The array spatial sensitivity encoding technique (a parallel imaging technique) was used to acquire imaging data by reducing geometric distortion for echo planar imaging (EPI). The anatomical three-dimensional (3D) spoiled gradient recalled echo (SPGR) sequence was obtained with a high-resolution 1-mm slice thickness (matrix size 256 × 256, field of view 256 × 256 mm, repetition time 6.9 ms, echo time 3 ms, flip angle 15°). T2*-weighted EPI sequence was used to measure BOLD contrast (repetition time 1,500 ms, echo time 25 ms, flip angle 70°, matrix size 128 × 128, field of view 192 × 192, in-plane resolution 1.5 × 1.5 mm^2^, 23 slices, 3-mm thickness, 0-mm space). A total of 303 volumes were collected over one session during the experiment in a sequential ascending order. A high-resolution T2 fast spin echo (T2 FSE) sequence (repetition time 4,300 ms, echo time 92 ms, matrix size 512 × 512, field of view 192 × 192, in-plane resolution 0.375 × 0.375 mm^2^, 23 slices, 3-mm thickness, 0-mm space) was obtained for the co-registration of 3D SPGR and EPI functional images. EPI functional images and T2 FSE structural images were acquired in an oblique coronal plane perpendicular to the long axis of the hippocampus. Almost the entire hippocampus (head, body, and tail) was included in the 23 slices. Functional images were localized in the sagittal plane of the SPGR image to identify the long axis of the hippocampus. Oblique coronal slices were fitted to the principal longitudinal axis of the hippocampus covering the entire bilateral medial temporal lobes. Firstly, distortions of fMRI signals were corrected by array spatial sensitivity encoding techniques, which were used to improve temporal and spatial resolution and reduce artifacts. Secondly, higher order shims were employed to directly compensate for local field distortions. These methods guaranteed homogeneity of the magnetic field.

#### Preprocessing and Estimations

Functional and structural MR images of the brain were preprocessed using the methods of realignment, temporal correlation, spatial normalization, and spatial smoothing. The data were analyzed using SPM8 software (Wellcome Trust Centre for Neuroimaging, University College London, London, UK). The first five volumes in each data set were removed to ensure that the signal reached a steady state. EPI functional images were corrected to account for the differences in slice acquisition times by interpolating the voxel time series using sinc interpolation and resampling the time series using the center slice as the reference point. The EPI functional images were then corrected for motion artifacts by realignment to the first volume. A mean EPI functional image was constructed during realignment. Co-registration was performed through two processes. Both the mean EPI functional image and the motion-corrected EPI functional images were co-registered to the T2 FSE structural image. The co-registered T2 FSE structural image was then co-registered to the structural SPGR image. Next, the registration points of the anterior and posterior horns of the lateral ventricle, top surface of the paracentral lobule, and bottom surface of the inferior temporal gyrus were checked in the T2 FSE structural, structural SPGR, and EPI functional images. Before spatial normalization, a parameter was produced by the segmentation process from the structural SPGR image. The structural SPGR image and EPI functional images were spatially normalized (1 × 1 × 1 mm) using the Montreal Neurological Institute space. Finally, the images were spatially smoothed using a Gaussian kernel with a full width at a half maximum of 3 mm. To detect the brain activation associated with a specific task while simultaneously reducing noise, the size of the smoothing kernel was kept at a recommended 2 to 3 times the voxel size [23]. A high-pass filter regressor (200 s) was included in the design matrix to exclude low-frequency noise and artifacts. To identify the correct activation spots of the brain, movement effects were discounted in a number of rows (298) and columns (3 translations and 3 rotations). For each subject, the three (new, lure, and repeated) regressors were estimated by a general linear model calculated by applying a canonical hemodynamic response function combined with time and dispersion derivatives. To assess the main effect of the lure images, as characterized by both the hemodynamic response function and these derivatives, an F-contrast obtained by the *F* test was required. Intra-individual activation maps were calculated by *F* tests. We calculated second-level group contrasts using a one-sample *t* test for each regressor (new, lure, and same) from the response of the canonical hemodynamic function. Differences in the intensities of the activation between task conditions were confirmed by a voxel-level threshold of *p* < 0.001 uncorrected, and a cluster-level threshold of FWE (family-wise error)-corrected *p* < 0.05. We extracted the average percent signal change values of the regions of interest (ROIs) from the anatomically defined AAL ROI atlas [24] and established 3D MRI atlases [25–27] for each subject and type of task stimuli using the MarsBar toolbox [28].

### Global Brain Connectivity Analysis

#### Subjects

Twelve patients with right cerebellar tumors (mean age 46.9 ± 13.3 years, range 17–66 years; 3 males, 9 females), 7 patients with left cerebellar tumors (mean age 53.3 ± 10.1 years, range 38–69 years; 2 males, 5 females), and 15 right-handed healthy volunteers (mean age 27.6 ± 6.5 years, range 20–44 years; 5 males, 10 females) were enrolled in this study. The normal healthy volunteers participating in the resting-state fMRI study were different from those included in the neuropsychological analysis and the event-related fMRI experiment. No participants had any signs or history of neurological or psychological diseases. This research was approved by the ethical committee of the University of the Ryukyus, and written informed consent was obtained from all participants.

#### Acquisition of Resting-State fMRI Data

Functional and anatomical images were obtained using a GE Discovery MR750 3.0 Tesla MRI scanner (GE Medical System) with a 32-channel head coil. In order to minimize head movement, the heads of each of the participants were fixed using foam pads. In order to reduce geometric distortion in EPI, a parallel imaging technique known as the array spatial sensitivity encoding technique was used during imaging data acquisition. T2*-weighted EPI images were used to measure BOLD contrast (repetition time 2,000 ms, echo time 30 ms, flip angle 70°, matrix size 64 × 64, field of view 256 × 256, in-plane resolution 4 × 4 mm, 42 slices, 4-mm thickness, 0-mm space). During EPI image scanning, participants were instructed to remain motionless, remain awake, relax with their eyes closed, and to try not to think about anything in particular. A total of 150 volumes were collected over one session in a sequential ascending manner (plus 5 initial discarded volumes). An anatomical three-dimensional spoiled gradient recalled echo (3D SPGR) sequence was obtained with high-resolution 1-mm slice thickness (matrix size 256 × 256, field of view 256 × 256 mm, repetition time 6.9 ms, echo time 3 ms, flip angle 15°). A high-resolution T2 fast spin echo (T2 FSE) sequence (repetition time 4,300 ms, echo time 92 ms, matrix size 256 × 256, field of view 192 × 192, in-plane resolution 1.33 × 1.33 mm, 42 slices, 4-mm thickness, 0-mm space) was obtained for the co-registration of 3D SPGR images and EPI functional images. EPI functional and T2 FSE structural images were acquired in an oblique axial transverse plane (tilted 30° anterior relative to the intercommissural plane).

#### Preprocessing and Analysis of Resting-State fMRI Data

Following this step, fMRI preprocessing, analysis, and visualization methods were conducted as implemented in SPM (8 package, http://www.fil.ion.ucl.ac.uk/spm8/) and the “conn” toolbox (www.nitrc.org/projects/conn). Images were corrected for slice acquisition time within each volume, motion corrected with realignment to the first volume, spatially normalized to the standard MNI EPI template, and spatially smoothed using a Gaussian kernel with a full width at half maximum of 8 mm. 3D SPGR images were co-registered with each mean EPI and T2 FSE image, and averaged together to permit anatomical localization of the functional connectivity at the group level. The transformed structural images were segmented into gray matter (GM), white matter (WM), and cerebrospinal fluid (CSF) using a unified segmentation algorithm.

In addition to removing noise correlations present in WM and CSF, the addition of six motion regressors (six realignment parameters and first derivatives) controlled for correlations due to movement. Data were filtered between 0.009 and 0.08 Hz.

#### Correlation Analysis with Global Brain Connectivity and the Lure Task

A map of GBC was computed from resting-state fMRI data using the “conn” toolbox (www.nitrc.org/projects/conn) [29, 30]. In the “conn” toolbox, correlation maps were calculated on the basis of seed-based correlation analysis using the AAL ROI atlas [24]. When the population correlation coefficient is zero, the distribution of correlation coefficient is consistent with the normal distribution. However, normal distribution of the correlation coefficient is lost when the correlation coefficient approximates to 1 [31]. Each ROI’s correlation coefficient map was transformed by Fisher’s *Z* transformation to *Z* value maps in order to normalize the correlation coefficient.

These *Z* value maps were averaged together across each subject in order to calculate GBC values. For correlation analysis of the GBC and score of the lure task, GBC values were extracted from the ROIs that were activated by the lure stimulus in the event-related fMRI experiment. The Pearson product–moment correlation coefficient was used to calculate correlations between GBC values and the correct response rates in the lure task. When the correlation coefficient was close to +1, the *r* value indicated a proportional connection between GBC values and the scores in the lure task (positive correlation). Conversely, when the correlation coefficient was close to −1, the *r* value showed an inverse proportion (negative correlation). We estimated the strength of the correlation in five categories: negligible correlation (0.00 to 0.30 or 0.00 to −0.30), low correlation (0.30 to 0.50 or −0.30 to −0.50), moderate correlation (0.50 to 0.70 or −0.50 to −0.70), high correlation (0.70 to 0.90 or −0.70 to −0.90), and very high correlation (0.90 to 1.00 or −0.90 to −1.00) [32].

### Statistical Analysis

The Kruskal-Wallis test for three independent samples or the Mann–Whitney *U* test for two independent samples was used to evaluate statistical significance in the neuropsychological assessments and fMRI behavioral tasks, since a normally distributed population could not be assumed. Preoperative and postoperative neuropsychological data were compared using the Wilcoxon signed rank test. Statistical significance was accepted at *p* < 0.05. The chi-square test was used to evaluate the performance in the block design subtest, since only two items of the block design subtest were evaluated as pass/fail.

## Results

### Preoperative Neuropsychological Profile of Patients with Benign Cerebellar Tumors

The results of the neuropsychological test of DST (*p* < 0.05), forward span of DS (*p* < 0.05), backward span of DS (*p* < 0.01), and total score of DS (*p* < 0.05) among patients with cerebellar tumors (*n* = 27) indicated a significant impairment as compared with the control group, which was further confirmed using the Mann–Whitney *U* test. To examine whether the profile of the impairments depended on the side of the lesion, patients with right and left tumors were compared to the control group. Performance on neuropsychological tests including MMSE, DST, forward span of DS, backward span of DS, total score of DS, and cube-copying test significantly differed across groups. Patients with right cerebellar tumors performed significantly worse than the control group on DST (*p* < 0.001), forward span of DS (*p* < 0.010), backward of DS (*p* < 0.001), and total score of DS (*p* < 0.05), as indicated by a post hoc Mann–Whitney *U* test with Bonferroni correction (Table 3). In contrast, there were no significant impairments in left-sided tumors as compared to the control group (Table 3). A Mann–Whitney *U* test was used to confirm that patients with right-side tumors showed significantly lower scores on the MMSE when compared with patients with left-side tumors (*p* < 0.05) (Table 3). No direct relationship was found between tumor size and scores on neuropsychological assessments, with the exception of the DST (*r* = −0.48, *p* < 0.05). None of subjects failed the fifth item of block design test. Chi-square analysis of the performance of the ninth item on the block design subtest revealed no significant difference between patients with right or left cerebellar tumors and the control group.

**Table 3:** Results of neuropsychological assessment of patients with damage to the right or left cerebellar hemispheres

Test	Cerebellar lesion		Controls, *n* = 23	*P*
	Right, *n* = 17	Left, *n* = 11		
Age, years	49.41 (13.99)	52.09 (9.48)	53.39 (14.05)	n.s
Education, years	13.29 (1.90)	12.82 (2.04)	12.91 (2.39)	n.s
Cerebellar lesion size, mL	8.99 (11.66)	9.65 (9.86)	–	n.s.
3MS	96.18 (4.37)	97.59 (2.52)	97.50 (2.14)	n.s.
MMSE	28.53 (2.00) †	29.73 (0.90)	29.13 (1.26)	0.034
WAIS-R Digit symbol test ^#^	8.65 (3.57)*	11.64 (3.38)	12.04 (2.44)	0.015
WAIS-R Digit span test				
Forward span	5.88 (1.05)*	6.45 (1.13)	6.86 (1.39)	0.025
Backward span	4.12 (0.86)*	4.64 (1.29)	5.43 (1.31)	0.006
Total score ^#^	10.06 (2.70)*	11.55 (2.30)	12.08 (2.82)	0.048
TMT, s				
Part A	36.94 (11.94)	34.82 (11.16)	30.74 (8.59)	n.s
Part B	64.82 (22.74)	64.00 (26.20)	52.52 (19.61)	n.s
Part B-A	27.88 (13.61)	29.18 (19.41)	21.78 (14.20)	n.s
Stroop test, s				
Reading (I)	24.94 (4.70)	22.00 (4.38)	22.78 (4.19)	n.s
Naming (II)	33.24 (6.89)	30.64 (5.28)	30.35 (6.59)	n.s
Interference (III)	58.41 (22.61)	48.09 (15.27)	48.04 (13.62)	n.s.
III-II	25.18 (18.46)	17.45 (12.19)	17.70 (8.19)	n.s.
Cube-copying test				
Point of connection	7.00 (1.97)	7.45 (1.04)	8.00	0.011
Plane-drawing errors	0.41 (1.00)	0.45 (0.82)	0	n.s

Values are mean (standard deviation). ^#^ denotes scaled score (mean = 10, standard deviation = 3). *P* indicates a significant difference after Kruskal-Wallis test or Mann–Whitney *U* test; * indicates a significant decline compared to controls (post hoc Mann–Whitney *U* test with Bonferroni correction, *p* < 0.05); † denotes a significant decline compared to patients with left lesions (post hoc Mann–Whitney *U* test with Bonferroni correction, *p* < 0.05)

*n.s.* not significant, *3MS* modified mini-mental state examination, *MMSE* mini-mental state examination, *TMT* Trail Making Test

### Postoperative Neuropsychological Profile of Patients with Benign Cerebellar Tumors

**Fig. 3:**
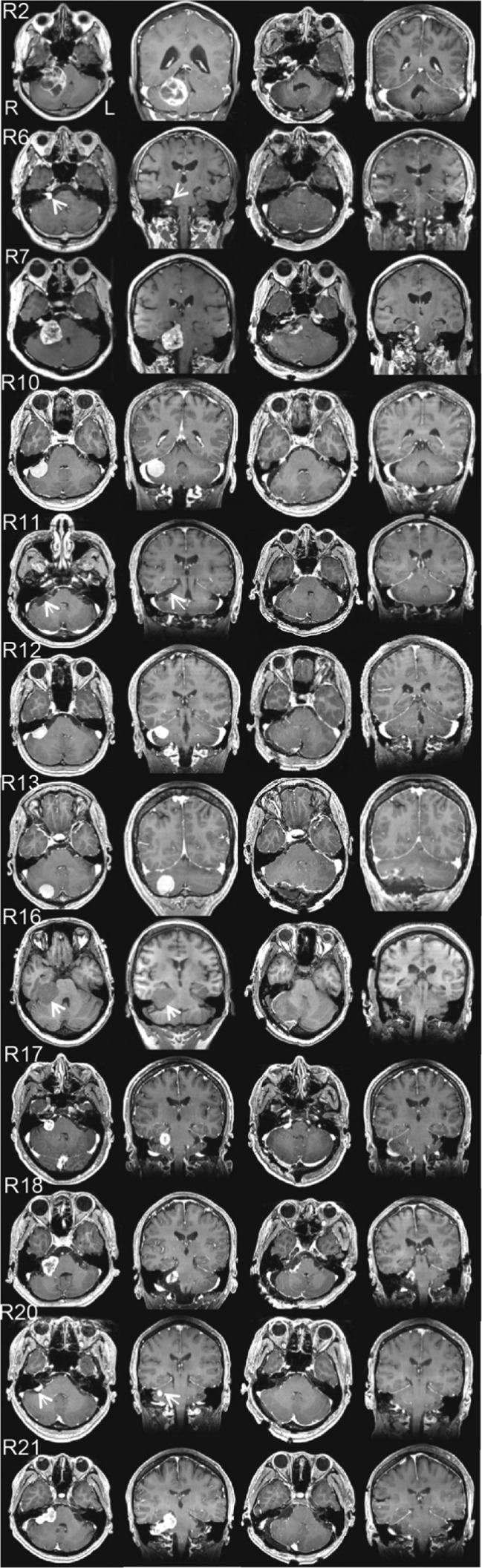
Panels of pre- and postoperative gadolinium-enhanced T1-weighted MRI of cases R2, R6, R7, R10, R11, R12, R13, R16, R17, R18, R20, and R21 (from *top* to *bottom*). *Rows* show axial and coronal images before the operation, and axial and coronal images after the operation (from *left* to *right*). Since Case R16 is subject to asthma, T1-weighted MRI was performed without contrast medium

Since most neuropsychological tests showed a significant decline in patients with right-sided tumors as compared with the control group at the preoperative stage, 12 patients with right cerebellar tumors were further tested 2 weeks to 18 months after resection of the tumor in order to investigate whether cognitive function became normalized after surgical decompression of the posterior lateral cerebellum. T1-weighted MRI confirmed that the decompressed cerebellum, especially lobule VI and Crus I had completely recovered after treatment (Table 2 and Fig. 3). Comparison of preoperative and postoperative neuropsychological tests revealed improvements in the raw scores of DST from 8.33 ± 3.20 to 8.92 ± 3.23, DS forward span from 6.08 ± 1.16 to 6.25 ± 1.60, DS backward span from 4.08 ± 0.90 to 4.58 ± 1.73, and DS total score from 9.92 ± 3.06 to 10.83 ± 4.59. However, no significant difference was found between neuropsychological assessments because of the small sample size (Table 4).

**Table 4:** Neuropsychological assessment of 12 patients with right cerebellar tumors at the preoperative stage and postoperative stage

Test	Preoperative assessment	Postoperative assessment	*P*
3MS	96.28 (4.99)	97.88 (2.59)	n.s.
MMSE	29.17 (1.11)	29.25 (1.76)	n.s.
WAIS-R Digit symbol test ^#^	8.33 (3.20)	8.92 (3.23)	n.s.
WAIS-R Digit span test			
Forward span	6.08 (1.16)	6.25 (1.60)	n.s.
Backward span	4.08 (0.90)	4.58 (1.73)	n.s.
Total score ^#^	9.92 (3.06)	10.83 (4.59)	n.s.
TMT, s			
Part A	33.58 (10.74)	36.50 (17.20)	n.s.
Part B	59.50 (23.33)	59.92 (23.91)	n.s.
Part B-A	25.92 (13.85)	23.42 (11.75)	n.s.
Stroop test, s			
Reading (I)	24.75 (2.30)	25.17 (3.79)	n.s.
Naming (II)	32.42 (4.64)	33.83 (7.76)	n.s.
Interference (III)	52.58 (18.92)	49.25 (14.64)	n.s.
III-II	20.17 (16.08)	15.42 (9.24)	n.s.
Cube-copying test			
Point of connection	7.33 (1.79)	7.92 (0.29)	n.s.
Plane-drawing errors	0.33 (0.89)	0	n.s.

Values are mean (standard deviation). ^#^ denotes a scaled score (mean = 10, standard deviation = 3). n.s. denotes not significant after a Wilcoxon singed rank test

### Hippocampal Function

**Fig. 4:**
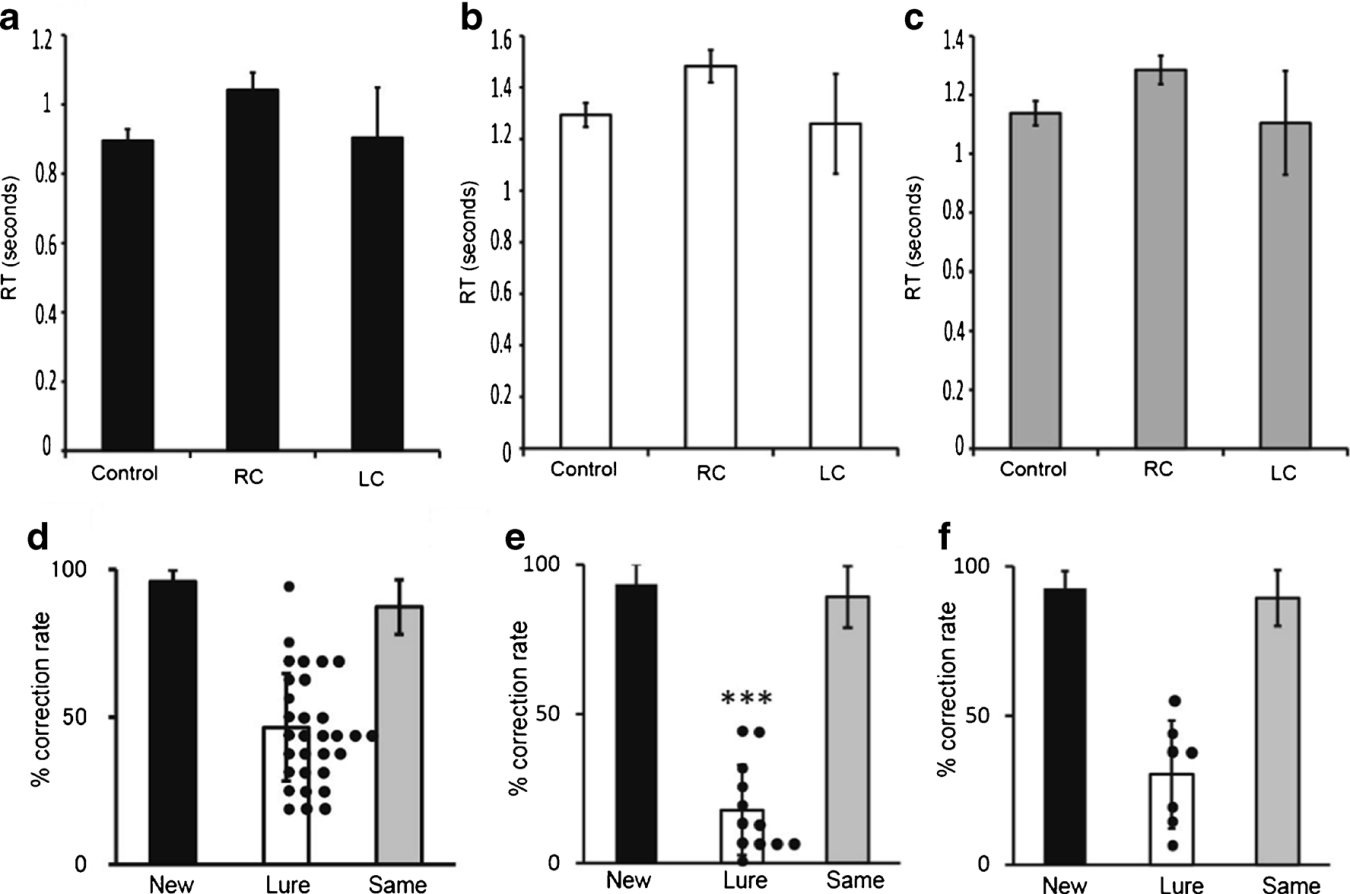
Pattern separation task examined by fMRI. **a**–**c**. Reaction times (RT) for new (**a**), lure (**b**), and repeated (**c**) tasks. Control indicates normal healthy volunteers (*n* = 30); RC, patients with right cerebellar tumors (*n* = 12); LC, patients with left cerebellar tumors (*n* = 7). *Bars* indicate mean; *Dots*, scores of individual cases. **d**–**f**. Percentage of correct response to new, lure, and repeated tasks in normal healthy volunteers (**d**), patients with right cerebellar tumors (**e**), and patients with left cerebellar tumors (**f**). *** in **e** indicates significant decrease compared to control (*p* < 0.001, Mann–Whitney *U* test)

Analysis of the reaction times for new, lure, and repeated task revealed no significant difference across groups (Fig. 4a–c). However, a significant decline was observed in the correct response rates during lure tasks in patients with right cerebellar tumors (13 ± 18 %; *n* = 12, age 53.4 ± 13.4 years) (*P* = 0.0003) compared with those of normal healthy volunteers (46.3 ± 3.3 %; *n* = 30, age 24.0 ± 5.2 years). Furthermore, no difference was found between patients with right and left cerebellar tumors (30 ± 18 %; *n* = 7, age 53.3 ± 10.1 years) (*P* = 0.25) (Fig. 4d–f).

### BOLD Responses

**Fig. 5:**
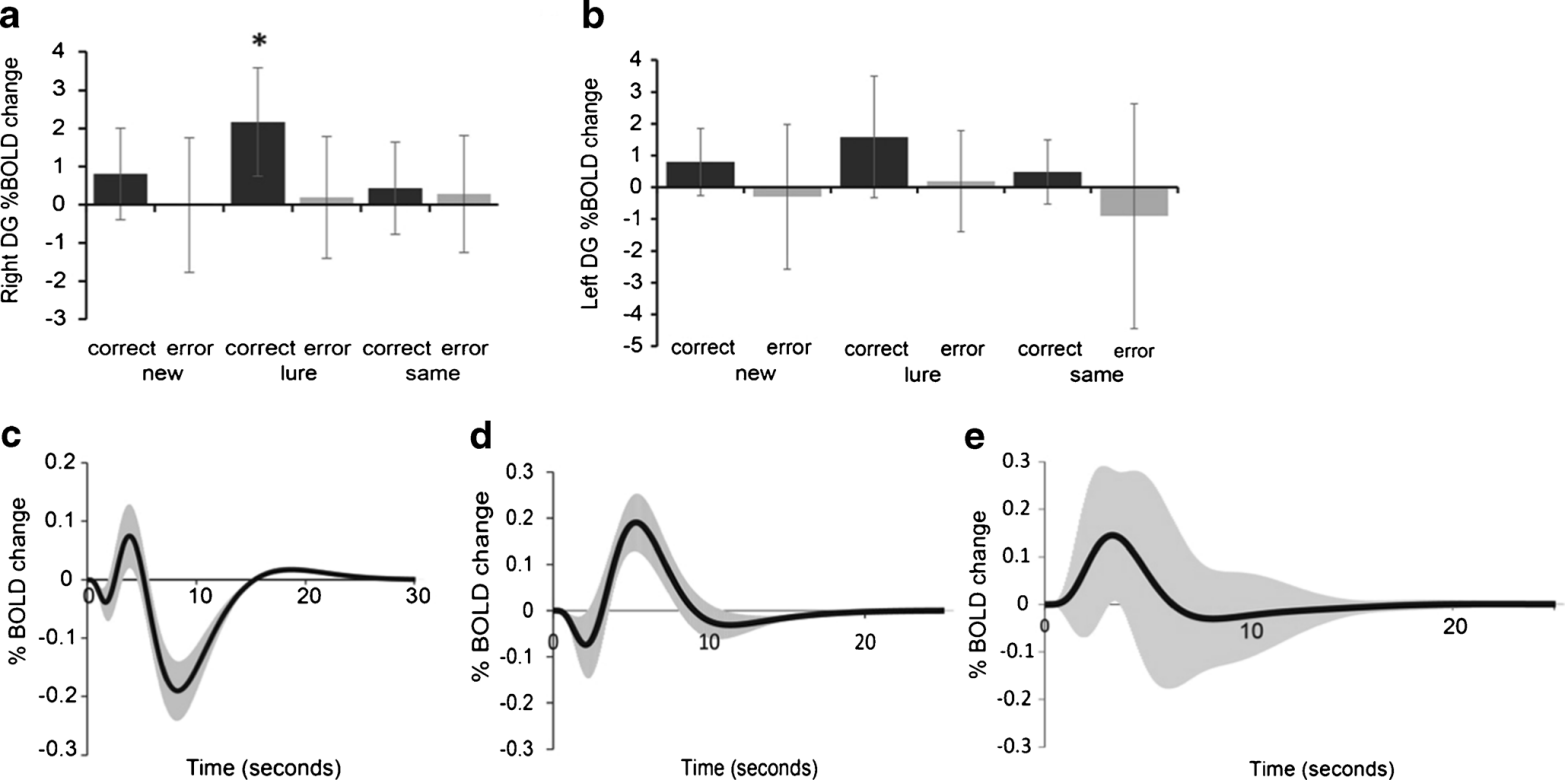
BOLD response by pattern separation task. **a**–**b**. *Bar graph* showing BOLD responses in the *right* and *left* DG for correct and error responses of the new, lure, and same tasks in normal healthy volunteers. Correct responses in the lure task were increased during activation of the right DG more so than correct and error responses in other tasks (*n* = 30) (two-way ANOVA, *F* = 4.52, *p* < 0.001, multiple comparisons two-sided test with Bonferroni-corrected critical *p* < 0.05) (**a**). There was no significant difference in % BOLD change in the left DG caused by responses and/or tasks (two-way ANOVA, *F* = 1.79, *p* = 0.13) (**b**). *y*-axis indicates the magnitude of the % BOLD change. * denotes a significant increase compared to other conditions (*p* < 0.05). *Graphs* showing the average BOLD curve for the lure task in normal healthy volunteers (**c**, *n* = 30), that of patients with right cerebellar tumors (**d**, *n* = 12), and that of patients with left cerebellar tumors (**e**, *n* = 7). The *black line* shows the average BOLD curve and the *gray line* shows standard deviation. The *x*-axis represents time course (s) of the percentage of BOLD change. The *y*-axis represents the magnitude of % BOLD change, or the percentage BOLD signal change from the resting to stimulus condition

We confirmed the BOLD signal activity in the right DG but not left ones correlated to correct response rate of the lure task rather than error one, nor other new and similar ones in normal healthy volunteers. These results indicate a crucial contribution of right DG for the performance of pattern separation ability (Fig. 5a, b). We therefore analyzed BOLD response patterns in the right DG during lure tasks. In normal healthy volunteers, the initial dip in the BOLD response occurred at 1.7 ± 1.3 s (mean ± S.D.) in time course, followed by a fractional increase in blood flow within 3.9 ± 4.2 s. The subsequent signal decrease was delayed by 8.3 ± 5.1 s, and the % BOLD change from that of the resting state was −0.19 ± 0.27, followed by a slope to a plateau or peak value for long pulses (>20 s) (Table 5 and Fig. 5c). Signal fluctuation or alteration of the BOLD pattern was found in patients with cerebellar tumors. A delayed latency of the initial positive peak (5.3 ± 1.9 s) with a large amplitude of % BOLD change (0.190 ± 0.060) subsequently followed by an initial dip (2.1 ± 0.9 s) was found in patients with right cerebellar tumors (Table 5 and Fig. 5d). For patients with left cerebellar tumors, we found a rapid initial peak (3.3 ± 1.4 s) without an initial dip, followed by a slope to a plateau value with a large S.D. value ranging from −0.18 to 0.28, indicating signal fluctuations among examinees of this group (Table 5 and Fig. 5e).

**Table 5:** Latency and amplitude of BOLD in the lure task in normal healthy subjects and patients with cerebellar tumors

Peak	Mean (SD) latency (second)			Mean (SD) amplitude (% BOLD change)		
	Normal (*n* = 30)	RC (*n* = 12)	LC (*n* = 7)	Normal (*n* = 30)	RC (*n* = 12)	LC (*n* = 7)
N1	1.7 (1.3)	2.1 (0.9)	0.2 (1.1)	−0.039 (0.165)	−0.074 (0.070)	0.000 (0.000)
N2	8.3 (5.1)	None	None	−0.191 (0.272)	None	None
P1	3.9 (4.2)	5.3 (1.9)	3.3 (1.4)	0.074 (0.292)	0.190 (0.060)	0.140 (0.140)

RC indicates patients with right cerebellar tumors; LC, patients with left cerebellar tumors. The first negative peak is defined as N1, second negative peak as N2, and first positive peak as P1. None indicates the absence of a peak

### Correlation Analysis with Brain Activation and Lure Task

**Fig. 6:**
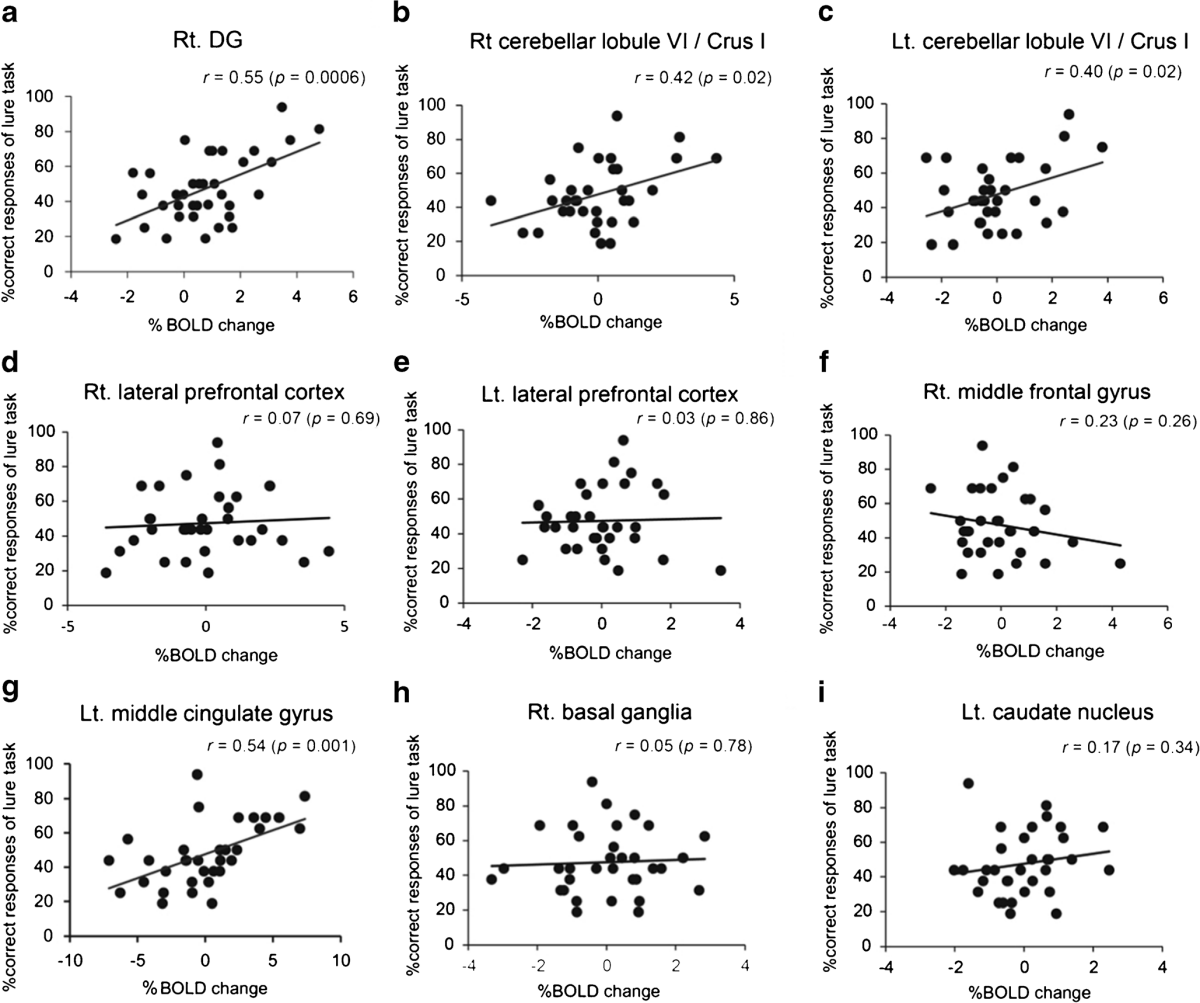
Regions related to pattern separation ability. Graph (**a** to **i**) showing correlation of percentage of correct responses of the lure task and that of BOLD signals in control subjects (*n* = 30). Hippocampus including dentate gyrus (DG) (**a**), right cerebellar lobule VI including Crus I (lobule VI/Crus I) (**b**), left lobule VI/Crus I (**c**), right lateral prefrontal cortex (**d**), left lateral prefrontal cortex (**e**), right middle frontal cortex (**f**), left middle cingulate gyrus (**g**), right basal ganglia (**h**), and left caudate nucleus (**i**). The percentage of correct responses in the lure task was significantly correlated with BOLD signals in right DG (**a**), right lobule VI/Crus I (**b**), left lobule VI/Crus I (**c**), and left middle cingulate cortex (**g**). No significant relationships were observed in the percentage of BOLD signals in the right and left lateral prefrontal cortex, right middle frontal cortex, right basal ganglia, and left caudate nucleus. The *r* value indicates the correlation coefficient of the Pearson product–moment (**a**–**i**). The *p* value indicates the significance level of the correlation coefficient. When the *p* value is lower than 0.05, the significance of correlation coefficient is accepted. *x*-axis represents the magnitude of % BOLD change that represents the rate of BOLD signal from stimulus to resting condition. *y*-axis represents the correct response rate in the lure task

**Fig. 7:**
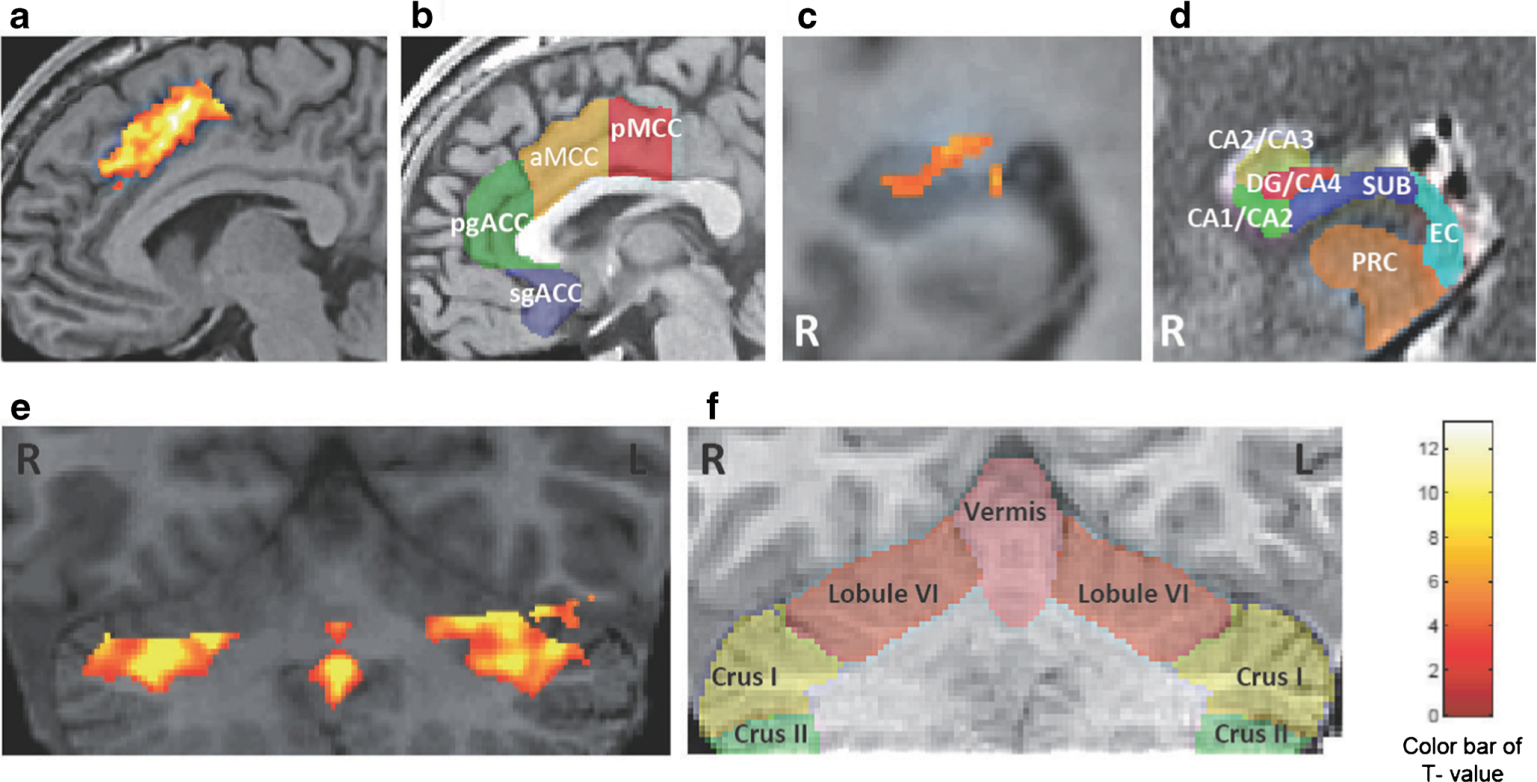
Identification of source ROIs in the activation maps and topographical schemas in healthy subjects. **a**–**f**. Identification of source ROIs in the activation maps during a lure task from healthy subjects (*n* = 30, voxel-level threshold at *p* < 0.001 uncorrected, corrected for multiple comparisons (family-wise error) to *p* < 0.05 using a cluster threshold) (**a**, **c**, and **e**). Each transparent color indicates the subdivisions of the rostral cingulate cortex (**b**), right hippocampus (**d**), and cerebellum (**f**). The color maps were painted according to references [24–27], and are explained in details in the text. **b**, *transparent red*, *yellow*, *green*, and *blue*, indicate the pMCC, posterior mid-cingulate cortex; aMCC, anterior mid-cingulate cortex; pgACC, pregenual anterior cingulate cortex; sgACC, subgenual anterior cingulate cortex, respectively. **d**, *transparent red*, *yellow*, *green*, *blue*, *light blue*, and *orange* indicate the hippocampal dentate gyrus and Cornu Ammonis 4 (DG/CA4); Cornu Ammonis 2 and 3 (CA2/CA3); Cornu Ammonis 1 and 2 (CA1/CA2); subiculum (SUB); entorhinal cortex (EC); and perirhinal cortex (PRC), respectively. **f**, *Transparent pink*, *orange*, *yellow*, and *green* indicate the cerebellar vermis, and bilateral lobule VI, Crus I, and Crus II, respectively. Anatomical identification is specified in the text. Note that the aMCC in **a**, and DG/CA4 in **c**, and lobule VI and/or Crus I in **e** were selectively activated. The *color bar* indicates *t* values (maximum *t* = 13.18, *white* represents the highest value). *R*, the right hemisphere. *L*, the left hemisphere

We began by detecting important regions for memory systems on the basis of the activation maps gathered from 30 healthy participants during the performance of a lure task involving pattern separation, which is an important human memory function of hippocampal circuits (Table 6). Next, we evaluated whether neural activity measured by local BOLD signal changes correlated to accurate response rates in the lure task across examinees, indicating a functional role of the regions instead of individual differences between examinees (Fig. 6a–i). The source ROIs were defined as the right DG, left anterior middle cingulate cortex (aMCC), and bilateral cerebellar lobule VI including Crus I, based on the correlation analysis of BOLD responses and percentage of correct responses in the lure task (Fig. 7a, c, e). Correct identification of source ROIs was confirmed by established 3D MRI atlases (Fig. 7b, d, f) [24–27]. Subdivisions of the rostral cingulate cortex, hippocampus, and cerebellum were painted on an individual structural SPGR image using FSLview in the FMRIB Software Library v5.0 (FMRIB Analysis Group, University of Oxford, Oxford, UK). The parameters of the GBC were extracted from the source ROIs above.

**Table 6:** Significant whole-brain activations for the lure task

Region	Cluster size	Peak of *T* value	Peak coordinates		
			*x*	*y*	*z*
Rt. cerebellar lobule VI/Crus I	10,064	13.18	−32	−47	−33
Lt. cerebellar lobule VI/Crus I	8,572	12.96	35	−54	−22
Lt. lateral prefrontal cortex	12,393	12.25	57	10	29
Lt. caudate nucleus	4,885	11.45	13	6	11
Lt. middle cingulate gyrus	19,034	11.07	3	11	42
Rt. lateral prefrontal cortex	8,725	10.74	−49	9	25
Rt. caudate nucleus	5,150	8.73	−13	11	2
Rt. hippocampus including DG	1,859	8.51	−20	−36	−6
Rt. middle frontal gyrus	1,927	7.32	−33	2	55

Voxel-level threshold at *p* < 0.001 uncorrected, corrected for multiple comparisons (family-wise error) to *p* < 0.05 using a cluster threshold

*Rt* right, *Lt* left, *DG* dentate gyrus

### Correlation with GBC and Pattern Separation

**Fig. 8:**
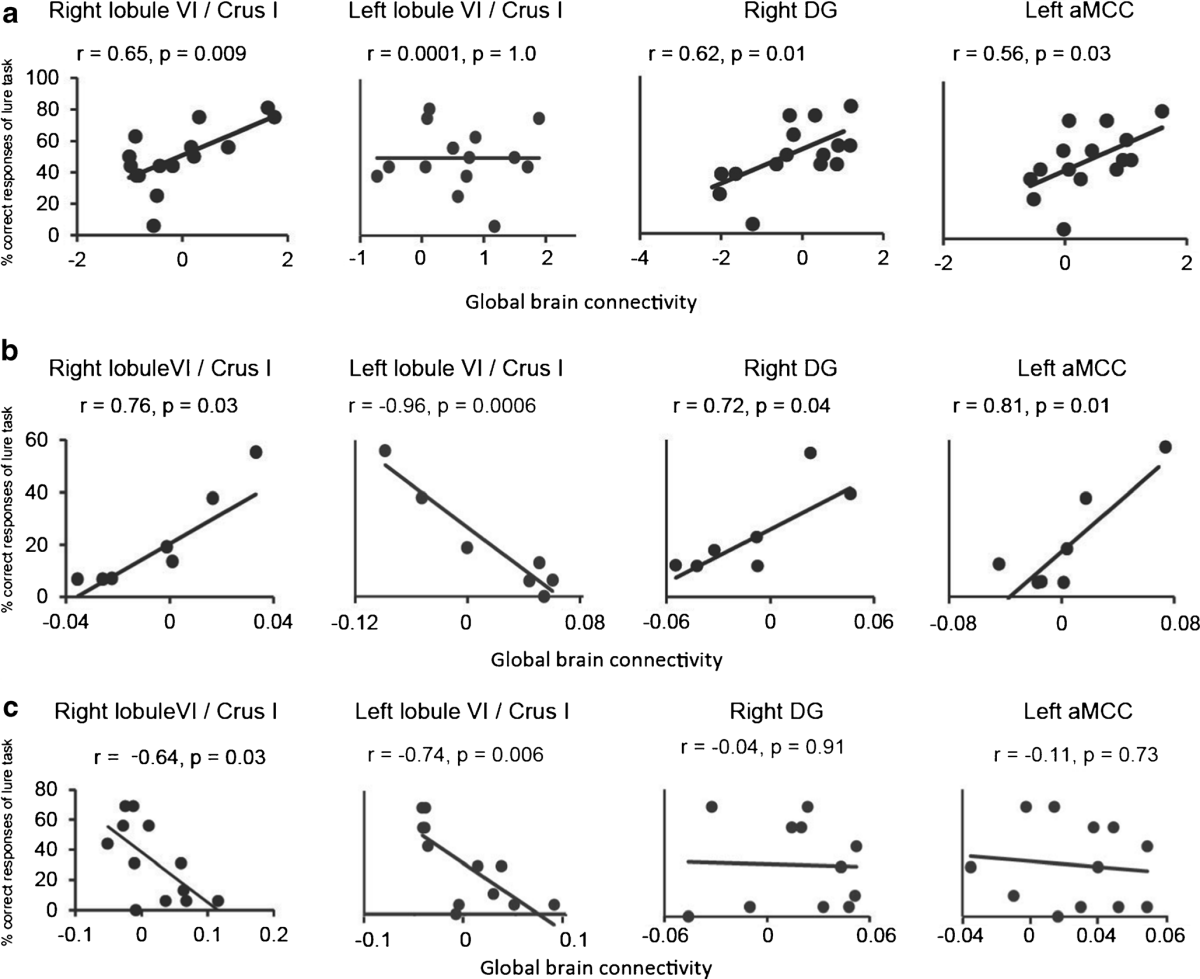
Correlation of GBC value and pattern separation ability. **a**–**c**. Graphs showing correlation between the correct response rate in the lure task and the GBC value in the right cerebellar lobule VI including Crus I (*lobule VI/Crus I*), left lobule VI/Crus I, right dentate gyrus (DG), and left anterior middle cingulate cortex (*aMCC*). **a** healthy subjects (*n* = 15). **b** patients with left cerebellar tumors (*n* = 7). **c** patients with right cerebellar tumors (*n* = 12). The *r* value, the correlation coefficient of the Pearson product–moment. The *p* value, the significance level of correlation coefficient. *x*-axis, the value of GBC. *y*-axis, the percentage of correct responses of lure task. Details are described in the text

For the GBC measure, we assessed standard resting-state fMRI data, and tested whether the right DG, left aMCC, and bilateral cerebellar lobule VI including Crus I had high GBC with the rest of the brain. The range of GBC values was from −2.1 to 1.9 in the normal control group, while patients with left and right cerebellar tumors showed more narrow ranges, from −0.09 to 0.07, and −0.07 to 0.11, respectively. The Pearson product–moment correlation coefficient was used to assess correlations between the GBC parameters of the four source ROIs and the percentage of correct responses in the lure task. The control subjects (*n* = 15) showed moderate positive correlations with the GBC of the right cerebellar lobule VI, including Crus I (*r* = 0.65, *p* < 0.01), right hippocampal DG (*r* = 0.62, *p* < 0.01), and left aMCC (*r* = 0.56, *p* < 0.05) (Fig. 8a), though no significant correlation was found in the left cerebellar lobule VI including Crus I (*r* = 0.0001, *p* > 0.05). We found that GBC connectivity correlated to correct response rates during lure tasks was limited to three regions including the right cerebellar hemisphere (lobule VI/Crus I), left aMMC, and right hippocampal DG. Herein, we raised the hypothesis that these three regions might play a crucial role in the human memory system, since rs-fMRI connectivity not only correlated to established structural connectivity, but also reflected well-known functional networks [33, 34]. Thus, GBCs in the right cerebellar hemisphere (lobule VI/Crus I), left aMMC, and right hippocampal DG were considered as the essential intrinsic connectivity of human cognition, statistically. In patients with left cerebellar tumors (*n* = 7), high positive correlations were found in the right cerebellar lobule VI including Crus I (*r* = 0.76, *p* < 0.05), right DG (*r* = 0.72, *p* < 0.05), and left aMCC (*r* = 0.81, *p* < 0.01) (Fig. 8b), while the left cerebellar lobule VI including Crus I showed an extremely negative correlation (*r* = −0.96, *p* < 0.001). In patients with right cerebellar tumors (*n* = 12), significant alteration of correlations were found in the right cerebellar lobule VI including Crus I (moderate negative correlation; *r* = −0.64, *p* < 0.05) and left cerebellar lobule VI including Crus I (high negative correlation; *r* = −0.74, *p* < 0.01), but no correlation was found in the right DG (*r* = −0.04, *p* > 0.05) or aMCC (*r* = −0.11, *p* > 0.05) (Fig. 8c). These results might collectively indicate an important cerebellar contribution to pattern separation.

## Discussion

We examined functional involvement of the posterior lateral cerebellum and its functional relationships with the hippocampus and the prefrontal cortex including anterior mid-cingulate cortex. Previous studies have investigated patients with cerebellar lesions such as tumors, strokes, and degenerative diseases, which frequently damage normal brain tissues. Our study evaluated patients with benign cerebellar tumors, because these lesions do not extensively destroy the surrounding normal brain tissues, so that neuropsychological assessment before and after treatment could clarify the functional involvement of the decompressed area. Human cerebellar cognitive function has been extensively studied in relation to the prefrontal cerebral cortex, but few studies have evaluated the correlation between hippocampal function and the cerebellar neocortex. Therefore, the present study also analyzed the functional activity of the posterior lateral cerebellum in relation to the hippocampus and anterior mid-cingulate cortex.

### Functional Involvement of the Posterior Lateral Cerebellum

To the best of our knowledge, the present study presents the first examination of the cognitive profiles of patients with benign cerebellar tumors that compress the posterior lateral cerebellum. Neuropsychological assessments indicated that patients with right cerebellar tumors showed impairments in working memory and psychomotor speed when compared with age-matched healthy controls. Patients with right cerebellar tumors also showed lower scores in MMSE than patients with left-sided tumors. We tried to interpret these cognitive declines with the view that the cerebellum contributes to intrinsic functional connectivity. The laterality and disease dominancy of cerebellar tumors may therefore be important. With regard to crossed cerebello-cerebral connections, patients with right-sided cerebellar lesion showed impairments in verbal tasks, whereas patients with left-sided tumors showed deficits in spatial tasks [35, 36]. Several studies have suggested a similar laterality in cognitive symptoms [37–39], and imaging studies have elucidated cerebellar topography and lateralization effects [40, 41]. Imaging studies [40, 41] have shown that activation peaks in language tasks were lateralized to the right lobule VI and lobule VII. In contrast, spatial processing showed greater left hemisphere activation, predominantly in lobule VI [40] and lobule VII [41]. Consistent with these imaging and clinical findings, Wang et al. [42, 43] reported cerebellar symmetry in relation to cerebral intrinsic functional connectivity. They indicated a right-lateralized cerebellar network including crus I/II and a portion of lobule VI, which couples to a left-lateralized cerebral network involving the inferior frontal gyrus, superior temporal gyrus, and temporal pole in the cerebral cortex. These regions include traditional language areas in the cerebral cortex, such that the cerebellar regions are commonly activated by language-related tasks. In our study, patients with right cerebellar tumors showed lower scores on the MMSE than patients with left-side tumors. Language processing is the main cognitive demand of the MMSE. Compression of the portions of the right lobule VI and Crus I connected with language areas alters the right-lateralized cerebellar network that supports language processing, which may lead to MMSE scores in patients with right cerebellar tumors. In the working memory test, lesion studies revealed that the inferior cerebellum was associated with performance on digit span test [13, 44]. There are laterality differences within the inferior lobe of cerebellum. The left inferior lobe of cerebellum is associated with the processing of aural information, whereas the right inferior lobe is involved in visual information [12]. Damage to left inferior cerebellar lobule VIII has been shown to reduce auditory digit span [44]. However, Ravizza et al. [13] revealed that performance on the auditory digit span test was unaffected by laterality of the damaged cerebellar hemisphere. Inconsistent with previous studies, the present study shows that patients with right-sided tumors exhibited impairment in the performance of the digit span test when compared with normal healthy volunteers. Chen and Desmond [11] hypothesized two cerebro-cerebellar networks for verbal working memory: the frontal/superior cerebellar network involving the right cerebellar lobule VI, Crus I, and Broca’s area, which is associated with articulatory rehearsal; and the parietal/inferior cerebellar network involving the right cerebellar lobule VIIB and inferior parietal lobe, which is related to maintenance/storage of information. In the present study, impairment on working memory in patients with right-sided tumors might be related to some change in the neural bases for processing verbal working memory caused by tumor compression in the right superior and inferior cerebellum. We also found that psychomotor speed was disturbed in patients with right cerebellar tumors compared with control subjects. DST is a psychomotor performance test thought to be affected by various cognitive demands, such as motor skill, attention, and visuomotor coordination [18]. Fronto-parietal cortical networks are related to performance on the DST, and these activations reflect the processes of visual searching and updating of working memory [45]. Since patients with right cerebellar tumors also exhibited impairment of working memory, the intrinsic functional connectivity between the left fronto-parietal network coupled with the right cerebellar hemisphere might be altered by compression. We suspected that the cognitive impairments of patients with right cerebellar tumors were related to alteration of cerebellar contributions to intrinsic functional connectivity. A huge tumor may secondary compress the dentate nucleus of the human cerebellar nuclei as well as direct compression of posterior lateral cerebellum. The nucleus conjuncted with neocortex and reported an important role for human learning and cognition [46].

We found an improvement in the raw scores of some of the neuropsychological tests after surgical intervention associated with anatomical normalization of the lateral posterior cerebellum. At the postoperative stage of neuropsychological estimation, some patients with no improvements in psychomotor speed showed transient neurological symptoms related to the IVth or VIth cranial nerve function. Double vision might be a factor in preventing optimal performance. However, follow-up neuropsychological assessments in patients with cerebellar lesions have been limited [39, 47]. These previous findings [39, 47, 48] might suggest that the cerebellum can recover from pathological insult by changing the relationships of cerebral connectivity. Further studies are required to identify the detailed mechanisms behind the restoration of cognitive function following treatment of cerebellar lesions.

### Hippocampal Memory Function in Patients with Cerebellar Tumors

The involvement of the cerebellum in non-declarative memory has been previously investigated. Patients with focal cerebellar lesions showed impaired motor sequencing [10]. Such investigations provide evidence for a cerebellar contribution to procedural learning and support the idea that the cerebellum is an important anatomical component for competent skill acquisition. However, it is still unclear whether cerebellar lesions influence hippocampal episodic memory. On the other hand, the role of the hippocampus in episodic memory has been extensively studied, and the DG subregion of the hippocampus is well known as a substrate for cognition [49].

Pattern separation is a function of the DG that transforms similar experiences or events into discrete, non-overlapping representations. The DG and its projections into the CA3 subregion have been shown to be involved in pattern separation [14]. fMRI was used to observe the process of pattern separation by scanning normal subjects during an incidental encoding task using pictures of common objects. The present fMRI study used a similar experimental paradigm to examine hippocampal memory function involvement in pattern separation in patients with cerebellar tumors. The BOLD response showed that the latency of the positive peak was significantly increased in patients with right cerebellar tumors, and these patients showed increases in the positive peak without any second negative peak. Logothetis et al. [50] reported a linear relationship between BOLD signals and neural activity. In addition, the BOLD signal was shown to represent the proportion of the cerebral blood flow (CBF) and the cerebral metabolic rate of oxygen (CMRO_2_) [51]. The factor of gain in the positive response was interpreted as either a reduction in CBF or an increase in CMRO_2_ [52]. Increases in BOLD responses are influenced by hemodynamics and metabolism based on the magnitude of neural response. However, the factor that exerts influence on the increase in positive BOLD responses has not yet been elucidated. Moreover, the physiological significance of the post-stimulus undershoot was interpreted as a normal decline to the resting state of neural activity [50, 53], and reductions in the second negative peak may reflect alteration of the neural responses in patients with right cerebellar tumors. The pattern of averaged BOLD responses did not illustrate the initial dip and post-stimulus undershoot in patients with left cerebellar tumors, which may have been masked by the initial dip and post-stimulus undershoot such that it could not be recognized by means of the extended standard derivation of BOLD responses. These results indicate fluctuations in the large BOLD signals in patients with left cerebellar tumors, though there was not much of a difference between patients with left and right tumors in the size of the lesion and the compressed portion of cerebellum. Further studies are required to examine the causal mechanism of fluctuation in the BOLD signals of patients with left cerebellar tumors.

These findings suggest that the cells surrounding a metabolic disturbance area may have provided appropriate assistance to the hippocampal circuitry. The assessment of hippocampal memory function indicated that patients with cerebellar tumors showed selective inability in a lure task, which reflects pattern separation inability and disturbance of the generating activity of young granule cells in the DG of the hippocampus. This inability was found in patients with both right and left cerebellar tumors, although performances in the other two tasks (new and same) were equal to those of normal healthy volunteers. Taken together, these findings indicate that the selective inability in the lure task was caused by cognitive dysfunction and not by motor impairment in patients with cerebellar tumors. Instead, cerebellar damage seems to affect hippocampal DG functions.

### Influence on Pattern Separation Function by Global Brain Connectivity of Posterior Lateral Cerebellum

The fMRI examination of cognitive processing observed cerebellar activity in the convergent area of the posterior lateral lobe, which also regulates smooth motor control. Activations of posterior lateral cerebellum were previously proposed as the internal model for new tools [7]. Our present GBC study demonstrated that the value was altered in patients with cerebellar tumors compared with the normal control group. Interestingly, the left and right values of patients with cerebellar tumors converged on a narrow window. It was reported that cerebellum, cingulate cortex, and hippocampus have high GBC values that are included in the top 10 % of GBC [29]. High GBC areas have more connectivity with cortical and subcortical regions [29]. The GBC values that were restricted within the narrow window may represent a reduction in connectivity induced by lesions to the posterior lateral cerebellum.

Our present study also showed that the right cerebellar lobule VI/Curs I, right DG, and left aMCC are important regions for pattern separation. In particular, patients with right cerebellar tumors showed a disruption in the correlation of GBC to these areas associated with pattern separation function. High GBC areas are believed to integrate cortical and subcortical activity and act as global hubs influencing cognitive control [29, 30]. According to resting-state fMRI analysis, it was reported that the posterior cerebellum has a functional connection with the prefrontal cortex, involving the anterior cingulate cortex for cognitive functions [8, 9]. Reduction in the GBC of patients with right cerebellar tumors not only elicited functional dissociation of the right and left lobule VI/Curs I from pattern separation ability but also affected the anterior mid-cingulate cortex and hippocampus. In light of these observations, global connectivity of the right posterior lateral cerebellum may play an important role in pattern separation as well as cognitive functions.

Interaction between the hippocampus and cerebellum occurs in the spatial domain [54]. Cerebellar impairment leads to dysfunction of the spatial cord as recorded by place cells in the CA1 hippocampus using L7-PKCI mice in which protein kinase C-dependent long-term depression at the parallel fiber-Purkinje cell synapses is blocked. Consequently, the cerebellum assists navigation by participating in the building of the hippocampal spatial map. Hippocampal-cerebellar interactions occur during spatio-temporal prediction [55]. Patients with right cerebellar tumors showed a high rate of error in the lure task, as was indicated by fMRI. Just as the cerebellum contributes to the fine tuning of coordination in skilled motor sequences in motor control, it also contributes to cognition by facilitating the precise discrimination of overlapping or similar experiences among episodic memories. Newly generated young neurons have been shown to facilitate pattern separation in the hippocampus [49]. Whether cognitive decline and disability in pattern separation in patients with cerebellar disease only reflect functional changes in new neurons or are instead associated with a decrease in hippocampal neurogenesis is an interesting question that requires further investigation.

## Conclusions

The present findings show that compression of the posterior lateral cerebellum causes impairment of cognitive function. Surgical decompression of the cerebellum facilitated cognitive recovery. The fMRI study demonstrated global connectivity between the Crus I, aMCC, and hippocampus during analysis of hippocampal memory function. The posterior lateral cerebellum acts as a global hub, cooperating with the hippocampus and anterior mid-cingulate cortex to facilitate pattern separation ability.
